# Biomarkers of tissue remodelling are elevated in serum of COVID-19 patients who develop interstitial lung disease - an exploratory biomarker study

**DOI:** 10.1186/s12890-024-03144-0

**Published:** 2024-07-10

**Authors:** Helene Wallem Breisnes, Diana Julie Leeming, Morten Asser Karsdal, Hannah Burke, Anna Freeman, Tom Wilkinson, Aishath Fazleen, Jannie Marie Bülow Sand

**Affiliations:** 1 Department of Biomedical Sciences, University of Copenhagen, Copenhagen, Denmark; 2 Hepatic and Pulmonary Research, Nordic Bioscience, Herlev, Denmark; 3 NIHR Southampton Biomedical Research Centre, University Hospital Southampton NHS Foundation Trust, Southampton, Hampshire England; 4 CES, Faculty of Medicine, University of Southampton, Southampton, Hampshire England

**Keywords:** COVID-19, ILD, Extracellular matrix, Biomarker, Neutrophil activity, Collagen

## Abstract

**Background:**

Coronavirus disease 2019 (COVID-19) is a viral pneumonia that can result in serious respiratory illness. It is associated with extensive systemic inflammation, changes to the lung extracellular matrix, and long-term lung impairment such as interstitial lung disease (ILD). In this study, the aim was to investigate whether tissue remodelling, wound healing, and neutrophil activity is altered in patients with COVID-19 and how these relate to the development of post-COVID ILD.

**Method:**

Serum samples were collected from 63 patients three months after discharge as part of the Research Evaluation Alongside Clinical Treatment study in COVID-19 (REACT COVID-19), 10 of whom developed ILD, and 16 healthy controls. Samples were quantified using neo-epitope specific biomarkers reflecting tissue stiffness and formation (PC3X, PRO-C3, and PRO-C6), tissue degradation (C1M, C3M, and C6M), wound healing (PRO-FIB and X-FIB), and neutrophil activity (CPa9-HNE and ELP-3).

**Results:**

Mean serum levels of PC3X (*p* < 0.0001), PRO-C3 (*p* = 0.002), C3M (*p* = 0.009), PRO-FIB (*p* < 0.0001), CPa9-HNE (*p* < 0.0001), and ELP-3 (*p* < 0.0001) were significantly elevated in patients with COVID-19 compared to healthy controls. Moreover, PC3X (*p* = 0.023) and PRO-C3 (*p* = 0.032) were significantly elevated in post-COVID ILD as compared to COVID-19.

**Conclusion:**

Serological biomarkers reflecting type III collagen remodelling, clot formation, and neutrophil activity were significantly elevated in COVID-19 and type III collagen formation markers were further elevated in post-COVID ILD. The findings suggest an increased type III collagen remodelling in COVID-19 and warrants further investigations to assess the potential of tissue remodelling biomarkers as a tool to identify COVID-19 patients at high risk of developing ILD.

## Introduction

Coronavirus disease 2019 (COVID-19) is a severe viral pneumonia that was estimated to be one of the leading causes of death in 2020 and 2021 with a global death count of 18.2 million [1]. The disease is caused by severe acute respiratory syndrome coronavirus 2 (SARS-CoV-2), an enveloped, single-stranded RNA virus which transmits through droplets and primarily infects the respiratory tract [2, 3]. Emerging data suggest multi-organ involvement where lung damage has been suggested as the most common serious manifestation [3, 4]. Long-term lung impairment may develop after clearance of the virus and in particular interstitial lung disease (ILD), often referred to as post-COVID ILD.

ILD is an umbrella term that refers to a wide range of inflammatory and fibrotic pulmonary diseases. Pulmonary fibrosis is characterized by a dysregulated extracellular matrix (ECM) remodelling, resulting in an excessive deposition of ECM components. The ECM is a highly dynamic, three-dimensional network present in all tissues, consisting of noncellular components such as elastin and collagens [5]. Collagens, specifically type I, III, and VI, constitute a major part of the lung tissue and contribute to the organ’s overarching architecture [6]. Type III collagen is the main protein produced during early tissue remodelling, followed by type I collagen [7, 8]. As the tissue remodels, ECM proteins are degraded and synthesized, resulting in the release of peptide fragments (neoepitopes) into circulation. In circulation, the fragments can be measured serologically to assess tissue remodelling. This has been done previously and shown to associate with disease severity, progression, and mortality in patients with pulmonary fibrosis [9–11]. Additionally, neoepitopes that has a bioactive function, called matrikines, have also been suggested to drive inflammation [12].

The lungs of COVID-19 patients have shown to be significantly altered with features such as diffuse alveolar damage, microthrombi in the interalveolar septa, and fibrotic changes [13–15]. Radiological and physiological lung abnormalities has been found in 71% of COVID-19 survivors three months after recovery [16] and in one third of patients abnormalities in pulmonary function was persistent at 12 months after discharge and radiological changes did not resolve in 24% of patients [17]. An interim analysis in the UK Interstitial Lung Disease Consortium (UKILD) post-COVID-19 study, estimated that the risk of residual lung abnormalities in patients with severe COVID-19 was up to 11% [18]. Infection with other coronaviruses such as middle east respiratory syndrome coronavirus (MERS-CoV) showed fibrotic abnormalities in one third of patients at 32 to 230 days follow up [19] and a long-term longitudinal study of SARS-CoV-infected health-care workers showed that 5% of the patients had residual interstitial fibrosis after 15 years [20].

Neutrophils have shown to play a prominent role in COVID-19 and increased blood neutrophil count, neutrophil-to-lymphocyte ratio, and elevated serum levels of neutrophil related cytokines have been observed in patients with severe disease [21–24]. Additionally, increased concentration of neutrophil extracellular traps (NETs) has been observed in tracheal aspirate, plasma, airways, and in the alveoli of SARS-CoV-2 infected patients [25–27]. In excess, NETs can contribute to a cytokine storm which may further exacerbate disease severity, progress into acute respiratory distress syndrome (ARDS), and lead to multiorgan failure and death [28, 29]. Indeed, elevated cytokine levels has been observed in COVID-19 patients and many are believed to have died from the response [30, 31]. The rapid increase in cytokines attract new inflammatory cells, resulting in an excessive infiltration of inflammatory cells in the lung tissue, further contributing to lung injury.

To explore aspects of the COVID-19 and post-COVID ILD pathophysiology in the non-acute phase, this study aimed to investigate the tissue remodelling, wound healing, and neutrophil activity using serological biomarkers.

## Materials and methods

### Study design and patients

Data and serum samples were collected as part of the Research Evaluation Alongside Clinical Treatment in COVID-19 (REACT COVID-19) study (20/HRA/2986) at University Hospital Southampton (UHS) NHS Foundation Trust (Southampton, United Kingdom) and the Southampton Research Biorepository (17/NW/0362) [32]. Data were collected from patients admitted to UHS with COVID-19 infection between May 2020 and January 2021. The patients were diagnosed at the point of attendance to the Emergency Department and admitted based on severity of symptoms and oxygen requirement.

Three months after in-hospital treatment and subsequent discharge, patients were followed-up for symptom re-assessment and serum samples were collected for research purposes. C-reactive protein (CRP), neutrophil count, and lymphocyte count were collected as part of clinical care. All patients provided informed written consent. Depending on patient-reported symptoms, further investigations such as pulmonary function tests and computed tomography (CT) of the thorax were undertaken. Demographic and clinical information was gathered from the UHS electronic clinical recording systems. Presence of ILD was determined by thoracic radiologists as part of clinical care and was defined as the presence of ground glass opacities, reticulations, or fibrosis within the lung parenchyma. As diagnosis was made during routine clinical management, the degree of ILD was not formally scored. Data on forced vital capacity (FVC) and diffusing capacity for carbon monoxide (DLCO) was registered for the post-COVID ILD patients. Comorbidities were registered for all patients and divided into five categories: diabetes, hypertension, obesity, respiratory diseases, or none. The respiratory diseases group included asthma, chronic obstructive pulmonary disease (COPD), and emphysema. Lastly, 16 unmatched healthy controls were obtained from a commercial vendor BioIVT (West Sussex, UK) and included in the analysis for comparison.

### Biomarker measurements

Serum samples collected from patients and healthy controls were stored at -80 °C until analysis. A panel of neo-epitope specific biomarkers were quantified in the collected serum samples, see Table 1. The biomarkers reflect type III collagen crosslinking (nordicPC3X™ [33]), type III and VI collagen formation (nordicPRO-C3™ [34] and nordicPRO-C6™ [35]), type I, III, and VI collagen degradation by matrix metalloproteinases (nordicC1M™ [36], nordicC3M™, [37] and nordicC6M™ [38]), calprotectin degradation by neutrophil elastase (nordicCPa9-HNE™ [39]), elastin degradation by proteinase 3 (nordicELP-3™ [40]), fibrin formation (nordicPRO-FIB™ [41]), and fibrin crosslinking and degradation (nordicX-FIB™ [42]). Biomarkers were quantified by enzyme-linked immunosorbent assays (ELISAs) utilizing neo-epitope specific monoclonal antibodies. In brief, streptavidin coated 96-well plates were incubated with appropriate biotinylated synthetic peptide or antibody for 30 min at 20 °C. Subsequently, 20 µL calibrator peptide, control, or serum sample was added to appropriate wells, followed by 100 µL monoclonal antibody targeting the specific sequence of interest or 80 µL buffer. The plates were incubated for 1–20 h at 4–20 °C according to the manufacturer’s instructions and where applicable followed by incubation with 100 µL secondary antibody for 1 h at 20 °C. Lastly, 100 µL TMB were added to the wells and incubated for 15 min before addition of 100 µL stopping solution, and plates were read spectrophotometrically, or 100 µL chemiluminescence substrate was added to the wells and incubated for 3 min before reading the relative light units using SpectraMax i3x. Biomarker levels were determined from the calibration curve. All incubation steps were performed with shaking at 300 rpm and followed by five washing steps. All samples were measured in duplicate with an acceptance criterion of coefficient of variance percentage (CV%) ≤ 20. Samples below the lower limit or above the upper limit of the measurement range (LLMR and ULMR) were given the value of the LLMR or ULMR for the respective assay.

**Table 1:** Biomarker panel

Biomarker	Description	Biological function
PC3X	Crosslinked pro-peptide of type III collagen	ECM formation and tissue stiffness
PRO-C3	Pro-peptide of type III collagen	ECM formation
PRO-C6	Pro-peptide of type VI collagen	ECM formation
C1M	MMP mediated degradation of type I collagen	ECM degradation
C3M	MMP mediated degradation of type III collagen	ECM degradation
C6M	MMP mediated degradation of type VI collagen	ECM degradation
PRO-FIB	Thrombin mediated degradation of fibrinogen	Wound healing
X-FIB	Plasmin mediated degradation of crosslinked fibrin	Wound healing
CPa9-HNE	Human neutrophil elastase mediated degradation of calprotectin	Neutrophil activity
ELP-3	Proteinase 3 mediated degradation of elastin	Neutrophil activity

ECM: extracellular matrix, MMP: matrix metalloproteinase

### Statistical analyses

Statistical analyses were performed using GraphPad Prism (version 9.1.2). Data were examined for normality using D’Agostino-Pearson omnibus test and Mann-Whitney test was used when comparing two groups. To investigate for correlations, Spearman’s rank correlation test was applied. Fisher’s exact test or Chi-square test was used to investigate contingency tables. Adjustments were done using a linear regression model in R (version 4.2.2). Data are shown as median with interquartile range, where differences were considered statistically significant if *p* < 0.05. Asterisks indicate the following: * = *p* < 0.05, ** = *p* < 0.01, *** = *p* < 0.001, **** = *p* < 0.0001, and ns = not significant.

## Results

### Patient characteristics

The 16 healthy controls were predominantly male (75%) and had a mean age of 42 years (standard deviation (SD) 16), see Table 2. 56% of the controls were Caucasian and 44% were Black. 56% of the 63 COVID-19 patients were male with a mean age of 56 years (SD 14). The age was statistically different between the healthy controls and COVID-19 patients (*p* = 0.005). 88% of the COVID-19 patients were Caucasian and 12% were Asian. Additionally, 19% had diabetes, 26% had hypertension, 13% had obesity, 13% had respiratory diseases, and 44% of the patients had no comorbidities. 16% of the COVID-19 patients had more than one comorbidity.

**Table 2:** Demographics of healthy controls and patients with COVID-19

Variables	Healthy controls	COVID-19	*p*-value
n	16	63	
Age (years), mean ± SD	42 ± 16	56 ± 14	*p* = 0.005
Male sex, n (%)	12 (75)	35 (56)	*p* = 0.254
Ethnicity, n (%)			-
Asian Black Caucasian	0 7 (44) 9 (56)	8 (12) 0 55 (88)	
Comorbidities, n (%)			-
Diabetes Hypertension Obesity Respiratory diseases None	- - - - -	12 (19) 16 (26) 8 (13) 8 (13) 28 (44)	

SD: standard deviation

In Table 3, demographics for patients with COVID-19 without ILD and COVID-19 with post-COVID ILD are shown. In short, 10 (16%) of the COVID-19 patients developed post-COVID ILD. The post-COVID ILD patients had nearly the same distribution of males as those without ILD (60% vs. 56%) and a slightly higher mean age (62 years vs. 55 years). There was no statistical difference in age, sex, ethnicity, comorbidities, CRP, neutrophil count, or lymphocyte count between the two groups. The FVC and DLCO was registered for 9 of the 10 post-COVID ILD patients, and they had a mean FVC of 82.7% (SD 24.3) and mean DLCO of 89.4% (SD 21.7) of predicted.

**Table 3:** Demographics of patients with COVID-19 no ILD and post-COVID ILD

Variables	COVID-19 without ILD	Post-COVID ILD	*p*-value
n	53	10	
Age (years), mean ± SD	55 ± 14	62 ± 10	0.204
Male sex, n (%)	30 (56)	6 (60)	> 0.999
Ethnicity, n (%)			0.330
Asian Caucasian	5 (10) 48 (90)	2 (20) 8 (80)	
Comorbidities, n (%)			0.792
Diabetes Hypertension Obesity Respiratory diseases None	11 (21) 12 (23) 7 (13) 7 (13) 23 (44)	1 (10) 4 (40) 1 (10) 1 (10) 4 (40)	
FVC (% predicted), mean ± SD	-	82.7 ± 24.3	-
DLCO (% predicted), mean ± SD	-	89.4 ± 21.7	-
CRP (mg/L), mean ± SD	88.0 ± 63.7	115.5 ± 32.3	0.095
Neutrophil count (x10^9^/L), mean ± SD	5.5 ± 3.3	9.5 ± 8.5	0.052
Lymphocyte count (x10^9^/L), mean ± SD	1.1 ± 0.6	1.3 ± 1.2	0.702

CRP: C-reactive protein, DLCO: diffusing capacity for carbon monoxide, FVC: forced vital capacity, ILD: interstitial lung disease, SD: standard deviation

### Tissue remodelling biomarkers are elevated in COVID-19 and post-COVID ILD

**Fig. 1:**
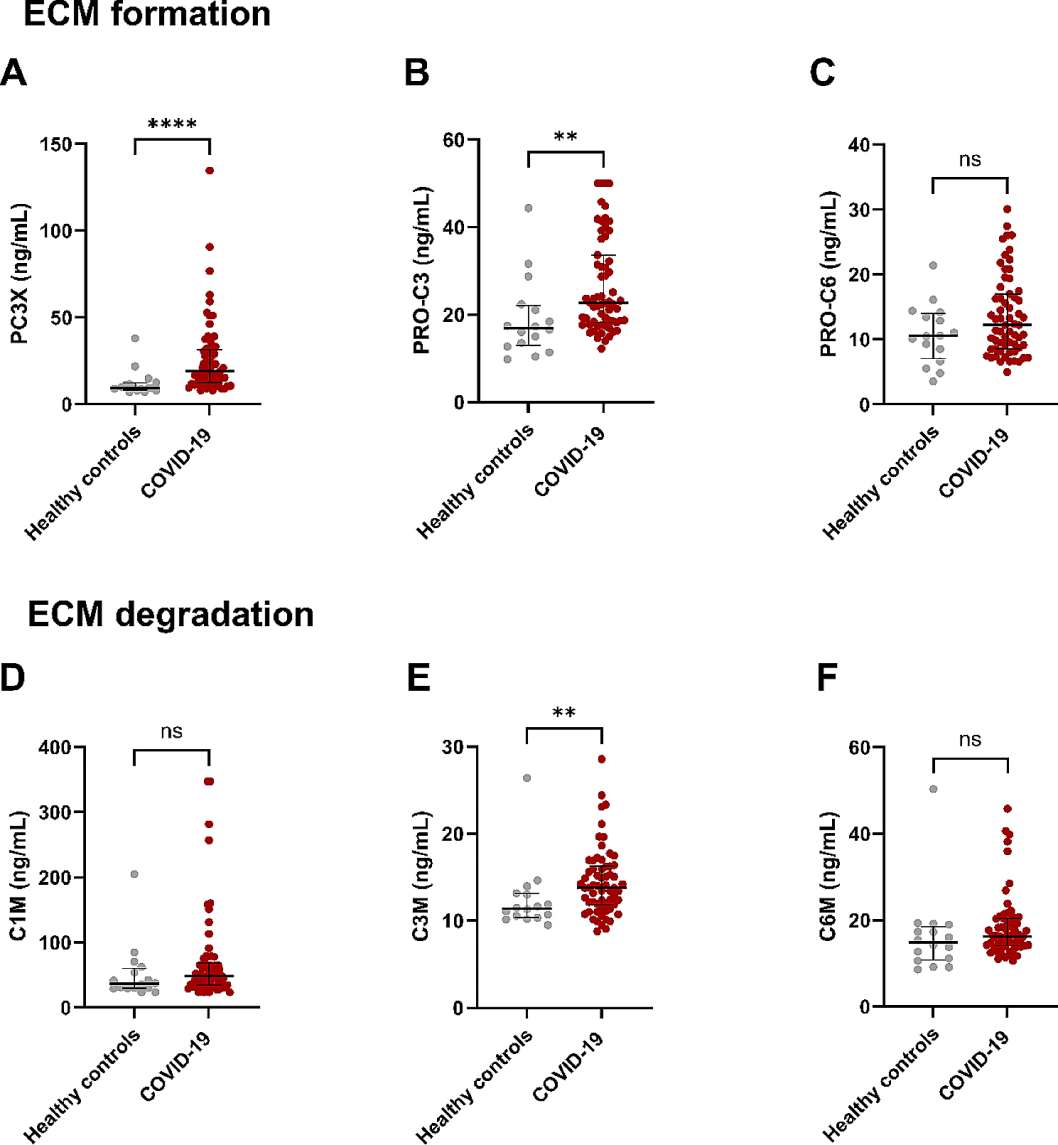
ECM formation and degradation biomarkers in patients with COVID-19. *Note* Healthy controls: *n* = 16, COVID-19: *n* = 63. Data were analysed with Mann Whitney test and shown as median with interquartile range

Biomarker data on healthy controls and patients with COVID-19 are summarized in Table 4. Patients with COVID-19 had significantly elevated serum levels of type III collagen formation biomarkers PC3X and PRO-C3 (*p* < 0.0001 and *p* = 0.002) when compared to healthy controls, see Fig. 1A and B. Type VI collagen formation biomarker PRO-C6 showed no significant difference between the two groups (*p* = 0.174, Fig. 1C).

The levels of type III collagen degradation biomarker C3M were significantly elevated in serum from patients with COVID-19 (*p* = 0.009), but type I and VI collagen degradation biomarkers C1M and C6M showed no significant difference in COVID-19 patients when compared to healthy controls (*p* = 0.107 and *p* = 0.084), see Fig. 1D, E and F.

**Fig. 2:**
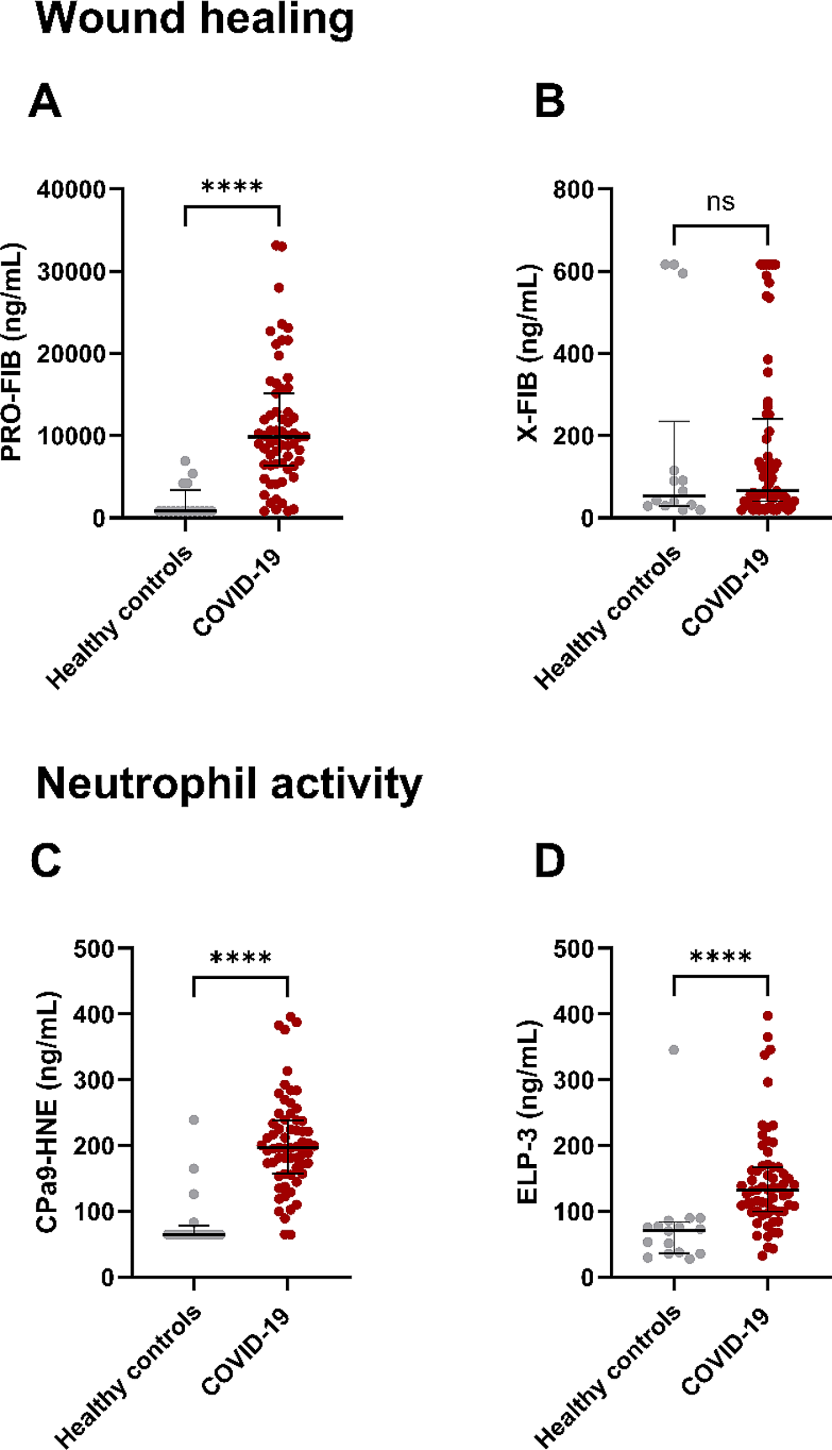
Wound healing and neutrophil activity biomarkers in patients with COVID-19. *Note* Healthy controls: *n* = 16, COVID-19: *n* = 63. Data were analysed with Mann Whitney test and shown as median with interquartile range

The levels of wound healing biomarker PRO-FIB were significantly elevated in serum of patients with COVID-19 when compared to healthy controls (*p* < 0.0001) whereas X-FIB did not show any difference between the two groups (*p* = 0.525), see Fig. 2A and B.

Neutrophil activity biomarkers CPa9-HNE and ELP-3 had significantly elevated serum levels in patients with COVID-19 when compared to healthy controls (both: *p* < 0.0001), see Fig. 2C and D. No correlation was found between neutrophil activity biomarkers and neutrophil count.

**Table 4:** Biomarker measurements in healthy controls and COVID-19

Biomarker	Healthy controls (ng/mL)	COVID-19 (ng/mL)	*p*-value
PC3X	9.6 [IQR 8.2–12.3]	18.9 [IQR 12.6–31.5]	***p*** ** < 0.0001**
PRO-C3	16.9 [IQR 12.9–22.1]	22.6 [IQR 18.2–33.6]	***p*** ** = 0.002**
PRO-C6	10.6 [IQR 7.1–14.0]	12.2 [IQR 8.6–17.0]	*p* = 0.175
C1M	36.7 [IQR 29.6–60.3]	48.2 [IQR 34.4–68.3]	*p* = 0.107
C3M	11.4 [IQR 10.4–13.1]	13.8 [IQR 11.9–16.3]	***p*** ** = 0.009**
C6M	14.8 [IQR 10.8–18.5]	16.2 [IQR 14.1–20.4]	*p* = 0.084
PRO-FIB	816 [IQR 816-3,368]	9,856 [IQR 6,368 − 15,152]	***p*** ** < 0.0001**
X-FIB	54.0 [IQR 29.3–235.0]	67.0 [IQR 40.2-241.1]	*p* = 0.525
CPa9-HNE	64.6 [IQR 64.6–78.6]	197.2 [IQR 157.3-238.5]	***p*** ** < 0.0001**
ELP-3	71.6 [IQR 36.2–83.9]	132.5 [IQR 99.9-167.3]	***p*** ** < 0.0001**

IQR: inter quartile range

**Fig. 3:**
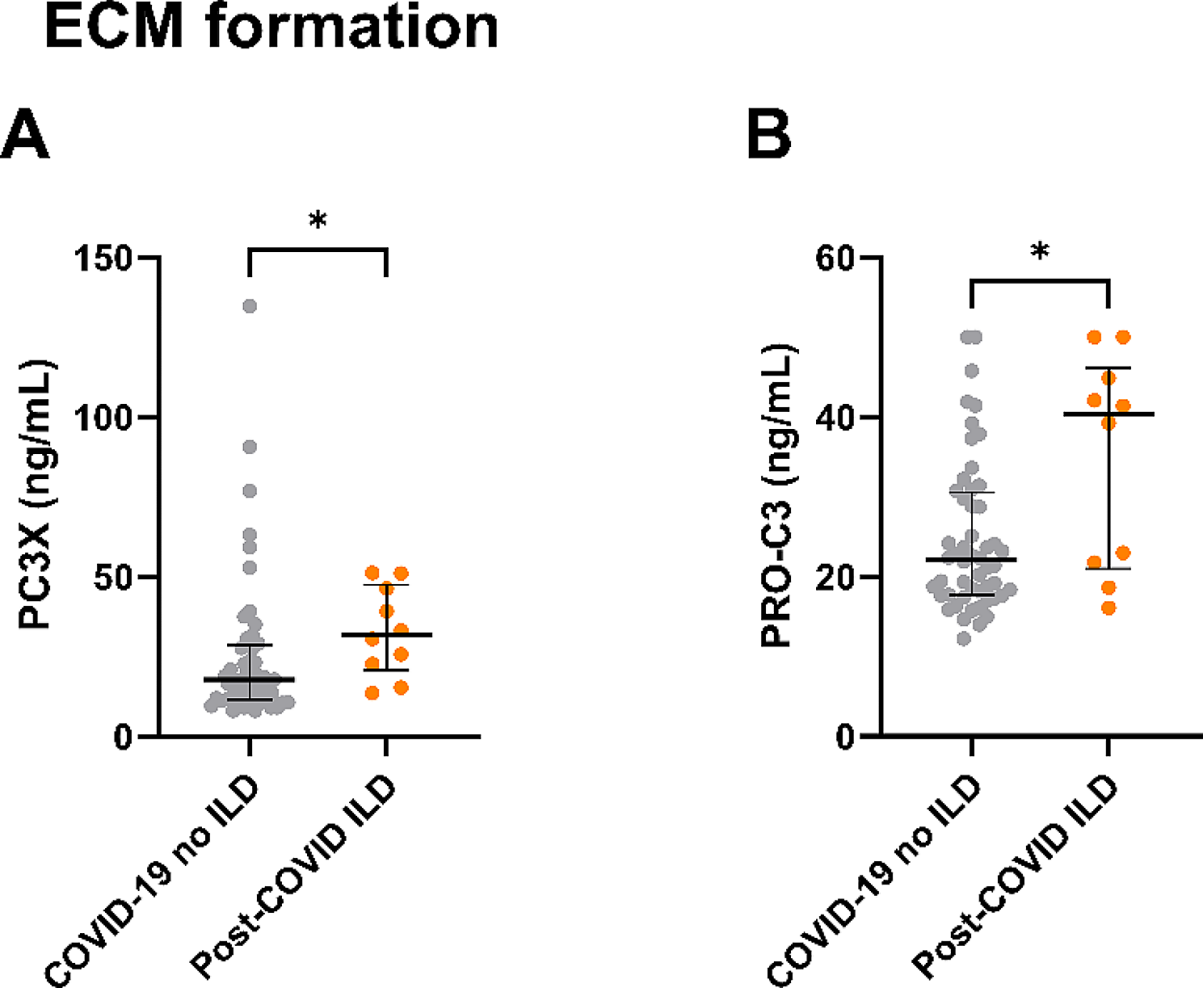
Type III collagen formation biomarkers in patients with COVID-19 and post-COVID ILD. *Note* COVID-19 without ILD: *n* = 53, post-COVID ILD: *n* = 10. Data were analysed with Mann Whitney test and shown as median with interquartile range

Biomarker data on patients with COVID-19 without ILD and COVID-19 with post-COVID ILD are summarized in Table 5. Type III collagen formation biomarkers PC3X and PRO-C3 showed significantly elevated serum levels in post-COVID ILD patients as compared to COVID-19 without ILD (*p* = 0.023 and *p* = 0.032, respectively), see Fig. 3. None of the other biomarkers showed significant differences between the two groups, though type VI collagen formation biomarker PRO-C6 was borderline significant with elevated serum levels in post-COVID ILD when compared to COVID-19 without ILD (*p* = 0.061).

**Table 5:** Biomarker measurements in COVID-19 without ILD and post-COVID ILD

Biomarker	COVID-19 without ILD (ng/mL)	Post-COVID ILD (ng/mL)	*p*-value
PC3X	17.8 [IQ 11.6–28.8]	31.9 [IQ 20.8–47.5]	***p*** ** = 0.023**
PRO-C3	22.1 [IQ 17.7–30.5]	40.3 [IQ 21.0-46.2]	***p*** ** = 0.032**
PRO-C6	11.3 [IQ 8.3–16.3]	18.7 [IQ 9.3–24.4]	*p* = 0.061
C1M	50.0 [IQ 36.1–67.6]	34.9 [IQ 27.5–64.9]	*p* = 0.121
C3M	13.8 [IQ 11.9–16.3]	14.2 [11.3–16.9]	*p* = 0.858
C6M	16.3 [IQ 14.2–20.2]	15.3 [IQ 12.5–20.4]	*p* = 0.617
PRO-FIB	9,644 [IQ 6,312 − 14,578]	12,250 [IQ 8,101 − 16,700]	*p* = 0.341
X-FIB	67.0 [IQ 33.5-205.8]	79.3 [IQ 39.0-443.5]	*p* = 0.600
CPa9-HNE	196.2 [IQ 167.8-231.4]	215.2 [IQ 142.5-268-4]	*p* = 0.615
ELP-3	132.7 [IQ 100.1-167.2]	115.2 [IQ 81.9–159.0]	*p* = 0.456

ILD: interstitial lung disease, IQR: inter quartile range

As the healthy controls and COVID-19 patients were non-matched, we investigated the influence of age, gender, ethnicity, and comorbidities on the biomarkers. Apart from PC3X, PRO-C6, and C6M, none of the biomarkers were influenced by these potential confounding factors in neither of the groups (data not shown). PC3X was significantly elevated in obese COVID-19 patients compared to non-obese COVID-19 patients (mean 36.4 [IQR 22.1–87.2] vs. 18.9 [IQR 12.7–31.9], *p* = 0.017). When excluding obese patients from the PC3X biomarker analysis no differences were observed compared to the original analysis (data not shown). Increasing PRO-C6 levels were associated with increasing age in both healthy controls and COVID-19 (correlation coefficient [r] = 0.329; *p* = 0.020 and *r* = 0.144; *p* = 0.002, respectively). When adjusting PRO-C6 for age, the results remained the same and there was no significant difference between the two groups (data not shown). Lastly, C6M levels were increased in Caucasians compared to Blacks in healthy controls (mean 17.3 [IQR 14.8–19.2] vs. 11.1 [IQR 9.2–13.8], *p* = 0.016). When comparing C6M levels in healthy controls and COVID-19 within Caucasian patients only, no differences were observed compared to the original analysis (data not shown).

## Discussion

This study aimed to investigate different pathophysiological aspects of COVID-19 and post-COVID ILD in the non-acute phase using exploratory biomarkers reflecting tissue remodelling, wound healing, and neutrophil activity. A panel of biomarkers reflecting these different biological processes were measured and showed different serum level signatures in healthy controls, COVID-19, and post-COVID ILD.

### Type III collagen remodelling is elevated in COVID-19

Following an acute injury to the lungs such as in COVID-19, the uncontrolled inflammatory response may lead to local and systemic tissue damage [43]. The tissue damage can be characterized by assessing the remodelling of various collagens, where type III collagen is one of the important fibrillar collagens that helps maintain the structure and function within various tissues [44]. PC3X, PRO-C3, and C3M are all biomarkers which measure different post-translational modifications leading to type III collagen remodelling. PC3X reflects ECM formation and tissue stiffness [33], PRO-C3 measures ECM formation, and C3M measures ECM degradation by matrix metalloproteinases. Both PC3X, PRO-C3 and C3M were shown to be significantly increased in serum of COVID-19 patients three months after discharge as compared to healthy controls. These findings align with Ackermann and colleagues who observed elevated PRO-C3 levels in plasma of severe COVID-19 patients when compared to no, mild, or moderate COVID-19, although no statistical significance was achieved in that study [45]. Changes in the remodelling of type III collagen have previously been associated with other diseases, such as liver and lung fibrosis [10, 11, 46]. These findings indicate an increased type III collagen remodelling in COVID-19 which could suggest similarities between the processes related to fibrotic diseases.

### Clot formation was elevated in COVID-19

Upon tissue injury, a cascade of events is initiated to repair the structural and functional integrity of the wound, including clot formation and clot resolution. The wound healing biomarker PRO-FIB measures a protein fragment which is released when fibrinogen is converted into fibrin and the biomarker X-FIB measures the plasmin mediated degradation of crosslinked fibrin; thus, the biomarkers reflect two distinct processes of wound healing, namely the clot formation and the resolution of a stable clot. Elevated levels of PRO-FIB were found in serum of patients with COVID-19, indicating that there was active wound healing with higher rate of clot formation at the time of blood sampling, three months after discharge, when compared to controls. Serum levels of X-FIB were not different between healthy controls and patients with COVID-19, indicating that resolution of the stable clot had not yet started or that the clot had not yet had enough time to become stable and create crosslinks. Another known predictor of severity and mortality in patients with COVID-19 is D-dimer. D-dimer is a protein fragment of fibrinogen and fibrin degradation that is released when a blood clot dissolves and it has been shown to be elevated in blood of severe COVID-19 patients at baseline and at three months follow-up [47, 48]. The contradictory findings between the D-dimer fragment and X-FIB could be explained by the fact that X-FIB measures the level of degraded crosslinked fibrin and thus reflects stable clot resolution whereas D-dimer reflects total clot resolution. This could indicate that COVID-19 patients have an abnormal wound healing process with an accelerated clot formation and subsequent breakdown of newly formed clots.

### Biomarkers of neutrophil activity are elevated in COVID-19

Previous studies have shown that severe COVID-19 patients have an increased blood neutrophil count, neutrophil-to-lymphocyte ratio, and elevated serum levels of neutrophil related cytokines, proposing an increased neutrophil activity [22–24, 27]. Thus, in this study, two neutrophil activity biomarkers, CPa9-HNE and ELP-3, were measured to investigate this biological process. CPa9-HNE reflects human neutrophil elastase mediated degradation of calprotectin and ELP-3 reflects proteinase 3 mediated degradation of elastin. Neutrophil elastase and proteinase 3 are both neutrophil serine proteinases which are released by neutrophils and play a part in the pathophysiology of various lung diseases [49]. Calprotectin is a protein mainly found within neutrophils, which gets released in the tissue together with neutrophil elastase when neutrophils initiate the process of NET formation [39]. Serum levels of CPa9-HNE and ELP-3 were significantly elevated in patients with COVID-19 as compared to healthy controls which indicate an increased neutrophil activity in patients with COVID-19 at three months after discharge. Neutrophil activity biomarkers did not correlate with neutrophil count, which was not surprising, as the biomarkers measure activity rather than the number of neutrophils. In other studies, calprotectin was found elevated in plasma of COVID-19 patients with severe disease as compared to those with mild [50] and neutrophil elastase was elevated in the blood of COVID-19 patients as compared to controls and associated with poor outcome and lung damage using CT score [51, 52]. Furthermore, the neutrophil activity in COVID-19 aligns with what has been seen in other lung diseases, such as COPD and ARDS, [53–55] and highlights the crucial function of neutrophil serine proteases and their possible pathological implications. The elevated serum levels of CPa9-HNE and ELP-3 align with these other studies and offer a tool to not only assess the neutrophil count, but neutrophil activity in patients with COVID-19.

### Post-COVID ILD was associated with increased type III collagen formation

Patients who developed post-COVID ILD had significantly elevated levels of the type III collagen biomarkers PC3X and PRO-C3 as compared to COVID-19 patients who did not develop ILD. These data indicate that type III collagen formation is increased even further in COVID-19 patients who develop ILD compared to those without ILD at three months after discharge. These findings align with other studies which have shown elevated PRO-C3 levels at baseline in patients with a fibrotic subtype of ILD, idiopathic pulmonary fibrosis (IPF), as compared to controls [10]. Additionally, PRO-C3 has been shown to be prognostic for IPF progression within 12 months and has been associated with a higher mortality at three-year follow-up in IPF patients with high baseline levels compared to low [9, 56]. Further investigations are needed to assess the potential of these biomarkers as tools to identify COVID-19 patients with a high type III collagen formation profile at risk of developing ILD. Such studies could potentially identify patients suitable for more in-depth investigations and these biomarkers may have the potential to be used for patient stratification or monitoring in clinical trials. As these biomarkers measure systemic levels of type III collagen remodelling, they could originate from any tissue or organ in the body. It can be speculated, however, if the increased type III collagen remodelling originates from the lung, considering the additional increase in serum levels associated with patients who develop post-COVID ILD.

A limitation of this study was the non-matched groups of healthy controls and COVID-19. However, with the exception of PC3X, PRO-C6, and C6M, biomarkers did not seem to be influenced by age, gender, ethnicity, or comorbidities. The biomarkers that had confounding factors were adjusted accordingly and no differences were found, indicating that our data are reliable. As data were collected as part of clinical care, a formal scoring for the severity of ILD was not conducted. In future studies, it would be interesting to investigate whether there are associations between ECM biomarkers and severity of ILD. Additionally, due to limited sample size, the findings might only be indicative of the pathophysiological processes that occurs in COVID-19 and post-COVID ILD and further investigations in bigger cohorts are needed to validate these findings.

## Conclusion

In this exploratory biomarker study, serological biomarkers were used to assess tissue remodelling, wound healing, and neutrophil activity in patients with COVID-19 and post-COVID ILD. Biomarkers of type III collagen remodelling, clot formation, and neutrophil activity were significantly elevated in COVID-19 compared to healthy controls, and type III collagen formation biomarkers were further elevated in patients with post-COVID ILD. These data align with previous findings and suggest that there is an increased neutrophil activity in patients with COVID-19 at three months after discharge, potentially leading to an excessive inflammatory response and subsequent lung tissue remodelling. Further investigations are needed to assess the potential of these biomarkers as tools to identify COVID-19 patients at high risk of developing ILD and to assist the development of new therapeutic approaches.

## Data Availability

No datasets were generated or analysed during the current study.

## Abbreviations

ARDS: Acute respiratory distress syndrome
COPD: Chronic obstructive pulmonary disease
COVID-19: Coronavirus disease 2019
CRP: C-reactive protein
CT: Computed tomography
CV: Coefficient of variance
DLCO: Diffusing capacity for carbon monoxide
ELISA: Enzyme-linked immunosorbent assay
FVC: Forced vital capacity
ILD: Interstitial lung disease
IPF: Idiopathic pulmonary fibrosis
IQR: Inter quartile range
LLMR: Lower limit measurement range
NET: Neutrophil extracellular trap
SARS-CoV-2: Severe acute respiratory syndrome coronavirus 2
SD: Standard deviation
ULMR: Upper limit measurement range
